# Biventricular Surgical Approach in Noonan Syndrome With Hypertrophic Obstructive Cardiomyopathy

**DOI:** 10.1016/j.jaccas.2025.106047

**Published:** 2025-11-25

**Authors:** Ryo Fujimoto, Shingo Hirao, Tatsuhiko Komiya

**Affiliations:** Department of Cardiovascular Surgery, Kurashiki Central Hospital, Kurashiki, Japan

**Keywords:** hypertrophic obstructive cardiomyopathy, left ventriculotomy, Noonan syndrome, right ventriculotomy, septal myectomy

## Abstract

**Background:**

Noonan syndrome is commonly associated with cardiac abnormalities such as pulmonary valve stenosis, hypertrophic cardiomyopathy, and atrial septal defect.

**Case Summary:**

A 19-year-old woman who was diagnosed with Noonan syndrome at birth had been receiving medical treatment for hypertrophic cardiomyopathy. The patient developed symptomatic hypertrophic obstructive cardiomyopathy, characterized by biventricular outflow tract obstruction. Surgical intervention was performed, including atrial septal defect closure and myectomy through left and right ventriculotomy approaches.

**Discussion:**

A right ventriculotomy was performed to resect the parietal band, septal myocardium, and anterior wall muscle. This was followed by a left ventriculotomy, enabling an extended septal myectomy from the resection margin near the aortic valve down toward the apex. This approach allowed for the removal of abnormal muscle bundles that were not visible through the aortic valve.

**Take-Home Message:**

When myectomy for hypertrophic obstructive cardiomyopathy is challenging, surgical access via both left and right ventriculotomy should be strongly considered.

## Graphical Abstract

**Figure:**
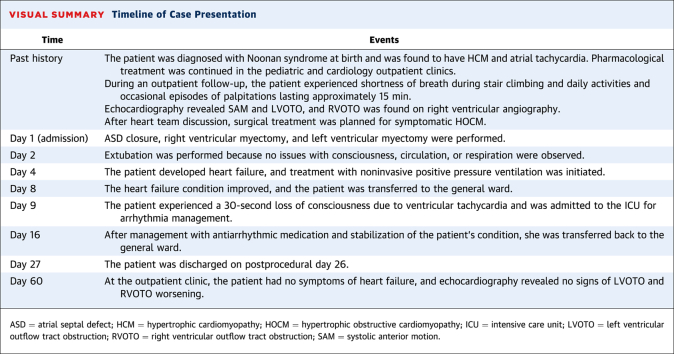

**Take-Home Messages**

• In cases of biventricular obstructive hypertrophic cardiomyopathy, when standard approaches are inadequate, a combined left and right ventricular incision approach should be considered.
• Comprehensive preoperative planning and anatomical understanding are essential, particularly regarding the bundle branches, to avoid postoperative conduction disturbances.

## History of Presentation

A 19-year-old woman with a known diagnosis of Noonan syndrome since birth presented at outpatient follow-up with exertional dyspnea and palpitations. The patient had a history of hypertrophic cardiomyopathy (HCM) affecting both the left and right ventricles and had been managed with pharmacological therapy for atrial tachycardia in pediatric and cardiology outpatient clinics. On admission, the patient's heart rate was 70 beats/min, and her systolic blood pressure was 130 mm Hg. Physical examination revealed a grade 3/6 systolic murmur, most prominent at the mitral valve area.

## Medical History

The patient was diagnosed with Noonan syndrome at birth based on the characteristic facial features, short stature, and presence of HCM. She had been receiving pharmacological treatment for HCM, atrial septal defect (ASD), and atrial tachycardia in pediatric and cardiology outpatient settings.

## Differential Diagnosis

Considering the patient's medical history along with findings from computed tomography (CT), right heart catheterization, and transthoracic echocardiography, 4 possible differential diagnoses were identified: Noonan syndrome, Noonan syndrome with multiple lentigines (ie, LEOPARD syndrome), cardiofaciocutaneous syndrome, and Costello syndrome.

## Investigations

The patient was determined as having NYHA functional class II status, presenting with symptomatic hypertrophic obstructive cardiomyopathy (HOCM). Transthoracic echocardiography revealed dynamic left ventricular outflow tract obstruction (LVOTO) with a mean pressure gradient of 64 mm Hg and moderate mitral regurgitation associated with systolic anterior motion (SAM) (Video 1) of the mitral valve. Furthermore, a mean pressure gradient of 52 mm Hg across the right ventricular outflow tract suggested right-sided pressure overload due to muscular subvalvular obstruction. A secundum-type ASD was also identified. Her B-type natriuretic peptide was elevated to 2,462 pg/mL. Right heart catheterization revealed a pulmonary artery pressure of 30 mm Hg, right ventricular pressure of 80 mm Hg, and pulmonary capillary wedge pressure of 15 mm Hg. A pressure gradient of 50 mm Hg between the pulmonary artery and right ventricle confirmed right ventricular outflow tract obstruction (RVOTO) (Video 2).

**Figure 1:**
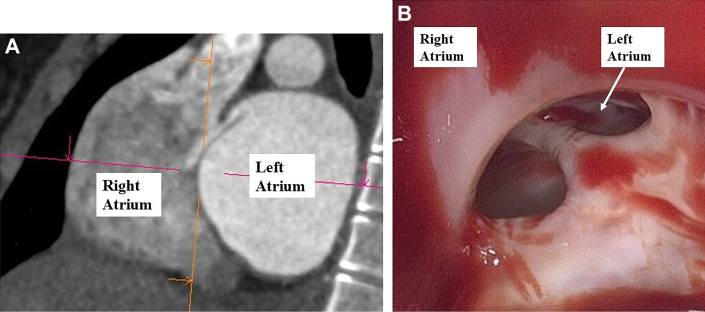
Preoperative and Intraoperative Images of the ASD (A) Preoperative CT showing the ASD. (B) Intraoperative inspection of the right atrium revealed the ASD with fibrous tissue components. ASD = atrial septal defect; CT = computed tomography.

Electrocardiography exhibited sinus rhythm, left axis deviation, abnormal Q waves, and T-wave inversion. CT revealed an 8 × 16 mm ASD at the fossa ovalis (Figure 1A) and biventricular myocardial hypertrophy. The septal thickness ranged from 20 to 23 mm, extending approximately 6 cm from the aortic valve. Considering the severity of the outflow tract obstructions and the presence of ASD, surgical intervention was planned to address the LVOTO, RVOTO, and ASD.

## Management

A median sternotomy was performed, and cardiopulmonary bypass was established with arterial cannulation of the ascending aorta and venous drainage via the superior and inferior vena cava. Upon opening the right atrium, a 10 × 20 mm secundum-type ASD was confirmed, along with a persistent valve-like structure over the foramen ovale, consistent with the preoperative CT findings (Figure 1B). The ASD was closed with direct suturing.

**Figure 2:**
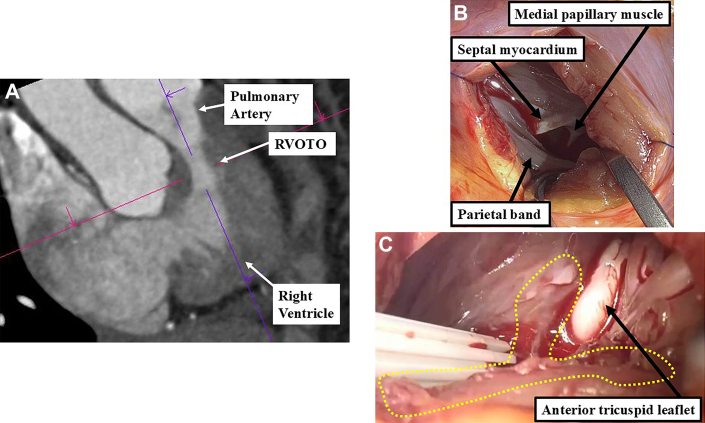
Findings Related to the RVOTO (A) Preoperative CT suggested a stenotic lesion below the pulmonary valve indicative of RVOTO. (B) The main causes of RVOTO were identified as the parietal band and hypertrophied interventricular septum. (C) Resection of the parietal band and anterior right ventricular wall relieved the RVOTO. CT = computed tomography; RVOTO = right ventricular outflow tract obstruction.

Through the tricuspid valve, the right ventricular outflow tract was visualized; however, no clear anatomical stenosis was observed, suggesting functional rather than structural RVOTO. Because of limited visibility, the approach was converted to a right ventriculotomy. Preoperative CT had identified the stenosis located 15 mm below the pulmonary valve (Figure 2A); therefore, the right ventricle was incised transversely at this location. The pulmonary valve itself appeared structurally normal, with no leaflet abnormality or valvular stenosis. The primary cause of the RVOTO was a markedly hypertrophied parietal band and adjacent septal myocardium, which appeared pale and fibrotic (Figure 2B). Although abnormal muscle bundles were present, no clear anatomical division of the right ventricle into separate chambers were observed, effectively ruling out a double-chambered right ventricle. To relieve the obstruction, additional muscle was resected along the anterior wall to a depth of approximately 5 to 8 mm (Figure 2C).

**Figure 3:**
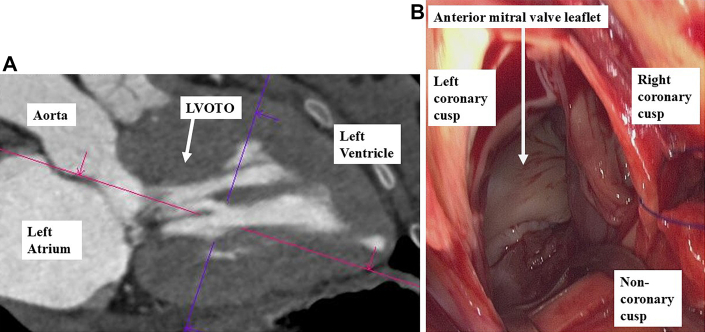
Findings Related to the LVOTO and Intraoperative View From the Aortic Valve Approach (A) Preoperative CT showing left ventricular hypertrophy and a suspected obstructive lesion causing LVOTO. (B) The aortic valve was rotated >30° clockwise, and the right noncoronary commissure was located far from the mitral valve. CT = computed tomography; LVOTO = left ventricular outflow tract obstruction.

Next, left atriotomy was performed to inspect the mitral valve, which appeared structurally normal. After closing the left atrium, an aortotomy was performed. Inspection through the aortic valve revealed extensive hypertrophy of the interventricular septum beyond the typical resection zone (Figure 3A).

The right coronary–noncoronary commissure was rotated approximately 30° clockwise from its expected orientation, positioning it farther from the mitral valve plane than normal (Figure 3B). The hypertrophied septum extended below the membranous septum, necessitating a broader resection to alleviate the LVOTO and SAM. To minimize risk to the left bundle branch located beneath the membranous septum near the noncoronary cusp, the resection began 10 mm below the membranous septum.

**Figure 4:**
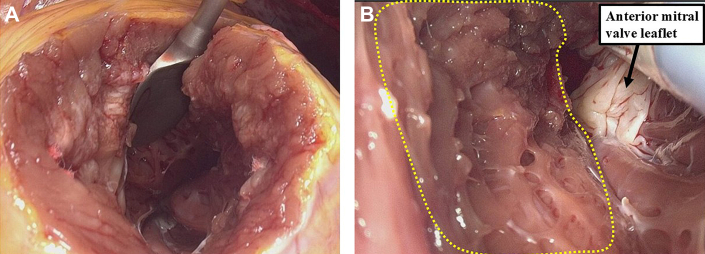
Intraoperative View From the Left Ventricular Apical Approach (A) Operative field after apical left ventricular incision. (B) Additional septal myectomy was performed by identifying the resection margin from the aortic valve side.

After aortic closure and reperfusion, intraoperative transesophageal echocardiography revealed residual SAM and inadequate septal resection. A second cardioplegic arrest was initiated, and a 4-cm left ventricular apical incision was made (Figure 4A). Through this access point, the margin of the initial resection was identified, and further septal tissue was removed. An aberrant muscle bundle connecting the lateral wall to the septum was also excised (Figure 4B).

The left ventricular incision was reinforced with felt strips and closed using 4-0 SH mattress sutures, followed by a continuous suture (Video 3). After weaning from cardiopulmonary bypass for the second time, the right ventricle-pulmonary artery pressure gradient improved to approximately 5 mm Hg, and the peak velocity in the left ventricular outflow tract was reduced to <2.0 m/s with the resolution of SAM.

The total volume of the resected myocardium was 8 mL from the left ventricle and 4 mL from the right ventricle. Histological examination confirmed features consistent with HCM.

## Outcome and Follow-Up

Postoperative echocardiography confirmed complete resolution of both LVOTO and RVOTO. SAM had resolved, and mitral regurgitation was reduced to a trivial level. A postoperative electrocardiogram revealed the development of a complete left bundle branch block. The patient's heart failure symptoms improved, and she was discharged on postoperative day 26.

Sixty days after discharge, follow-up transthoracic echocardiography revealed no recurrence of LVOTO or RVOTO. The patient remained asymptomatic in daily life and had improved to NYHA functional class I.

## Discussion

Noonan syndrome is a genetic disorder characterized by abnormal signaling in the RAS/MAPK pathway. It presents distinct facial features, short stature, and congenital heart defects. Disorders with similar genetic mutations and overlapping clinical features are collectively referred to as RASopathies.

Cardiovascular involvement occurs in approximately 80% of individuals with Noonan syndrome, with pulmonary valve stenosis in 57%, secundum-type ASD in 32%, and HCM in 16%.^1^ HCM in this syndrome often presents early and tends to be more severe.^2^ The current case involved a patient with HOCM and ASD.

Current guidelines recommend considering septal reduction therapy for adults with HCM who remain symptomatic (NYHA functional class ≥II) despite treatment with beta-blockers or calcium-channel blockers. Additional options include myosin inhibitors, disopyramide, or septal reduction procedures.^3^ Although myosin inhibitors are effective in adult HOCM,^4^ their utility in syndromic cases such as Noonan syndrome with biventricular outflow tract obstruction (BVOTO) have not yet been established.

Surgical intervention may be warranted in rare cases of BVOTO if both LVOTO and RVOTO reach clinical thresholds. LVOTO is typically an indication for surgery if the peak gradient is ≥50 mm Hg with symptoms.^3^ RVOTO is considered for surgery in symptomatic patients with a right ventricular systolic pressure of ≥80 mm Hg.^5^ When both obstructions are significant, simultaneous surgical relief is recommended.^6^

The standard approach for relieving LVOTO is transaortic septal myectomy. However, when septal thickening extends deeply or toward the apex or right ventricle, this approach may be insufficient. In the current case, the aortic valve was rotated approximately 30° clockwise, making exposure difficult via the aortic route. The residual SAM after the first resection confirmed the need for a left ventricular apical approach, which successfully eliminated the obstruction.

Preoperative imaging is crucial for understanding anatomical variations. In this case, the right noncoronary commissure was rotated and displaced from the mitral valve plane. Myectomy extended below the membranous septum, increasing the risk of left bundle branch damage. Therefore, understanding the conduction system's anatomy is crucial for minimizing such risks. Septal myectomy was extended close to the right fibrous trigone, but care was taken to stay away from the membranous septum to avoid injury to the His bundle and the central portion of the left bundle branch. However, because of the high risk of damaging peripheral branches of the left bundle, the patient developed complete left bundle branch block on postoperative electrocardiography.

The right bundle branch courses through the muscular septum beneath the medial papillary muscle and extends to the right ventricular endocardium and moderator band. Avoiding resection below the medial papillary muscle helped preserve right bundle function.

This case highlights the effectiveness of a biventricular surgical approach—incisions into both the right and left ventricles—for complex BVOTO. Previous reports support RVOTO muscle resection through ventriculotomy,^7^ and although left ventricular apical incisions are typically avoided, they are justified for deep septal lesions.^8^ Unlike sarcomeric HCM, HCM in Noonan syndrome often features disorganized myocytes and interstitial fibrosis,^9^ necessitating more extensive resection.

## Conclusions

Although standard myectomy approaches typically involve the aortic, pulmonary, or transvalvular routes, direct incisions into the left and right ventricles should be considered when adequate resection cannot be achieved through conventional methods. A thorough understanding of the cardiac conduction pathways, including the right and left bundle branches, is crucial to avoid iatrogenic conduction defects during extensive muscle resection.

## Funding Support and Author Disclosures

The authors have reported that they have no relationships relevant to the contents of this paper to disclose.

## Appendix

Video 1 Preoperative TTE Revealed Left Ventricular Hypertrophy, LVOTO, SAM, and Moderate Mitral Regurgitation

Video 2 Right Ventricular Angiography Exhibited Findings Consistent With RVOTO

Video 3 ASD Closure, Right Ventricular Myectomy, and Left Ventricular Myectomy Were Successfully Performed
